# Communication and Computing Task Allocation for Energy-Efficient Fog Networks

**DOI:** 10.3390/s23020997

**Published:** 2023-01-15

**Authors:** Bartosz Kopras, Filip Idzikowski, Bartosz Bossy, Paweł Kryszkiewicz, Hanna Bogucka

**Affiliations:** Faculty of Computing and Telecommunications, Poznan University of Technology, 60-965 Poznań, Poland

**Keywords:** fog network, energy efficiency, latency, cloud, edge computing

## Abstract

The well known cloud computing is being extended by the idea of fog with the computing nodes placed closer to end users to allow for task processing with tighter latency requirements. However, offloading of tasks (from end devices to either the cloud or to the fog nodes) should be designed taking energy consumption for both transmission and computation into account. The task allocation procedure can be challenging considering the high number of arriving tasks with various computational, communication and delay requirements, and the high number of computing nodes with various communication and computing capabilities. In this paper, we propose an optimal task allocation procedure, minimizing consumed energy for a set of users connected wirelessly to a network composed of FN located at AP and CN. We optimize the assignment of AP and computing nodes to offloaded tasks as well as the operating frequencies of FN. The considered problem is formulated as a Mixed-Integer Nonlinear Programming problem. The utilized energy consumption and delay models as well as their parameters, related to both the computation and communication costs, reflect the characteristics of real devices. The obtained results show that it is profitable to split the processing of tasks between multiple FNs and the cloud, often choosing different nodes for transmission and computation. The proposed algorithm manages to find the optimal allocations and outperforms all the considered alternative allocation strategies resulting in the lowest energy consumption and task rejection rate. Moreover, a heuristic algorithm that decouples the optimization of wireless transmission from implemented computations and wired transmission is proposed. It finds the optimal or close-to-optimal solutions for all of the studied scenarios.

## 1. Introduction

### 1.1. Motivation

Fog, loosely defined as “a cloud closer to the ground” [1] or “an extension, not a replacement, of the cloud” [2], is a computing and networking paradigm that aims to bring computational, storage and networking resources close to the edge of the network [3]. It provides access to these resources through geographically distributed FN.

**Figure 1:**
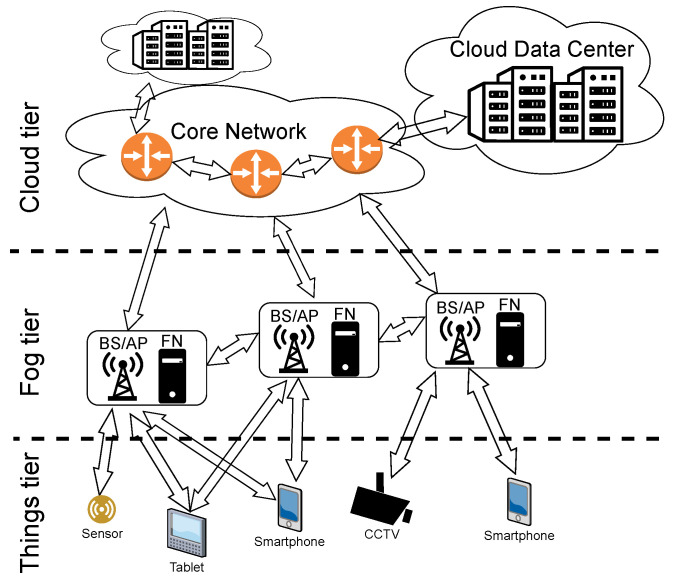
Example fog network architecture.

A fog network can be used for offloading computational tasks from end users to other nodes in the network. Energy and time spent on transmission can be saved when information is processed in one of the nearby FNs rather than in the remote cloud DC. However, these cloud DCs are expected to be more energy-efficient in terms of computation due to their scale (Google, for example, reports that its cloud services are carbon neutral [4]). How shall computation tasks be distributed over the computation nodes then? We take a holistic view on modeling and optimizing costs related to offloading in this work. Wired and wireless networks are covered starting from the end users, going through the FN, the core network and ending at the cloud. An example of such a network, divided into tiers, is shown in Figure 1.

An example scenario where computational resources provided by FN can be used to efficiently process information is controlling and predicting air quality [5]. Multiple sensors with limited computational capacities can send required data to nearby FN.

### 1.2. Related Work

Previous research on task allocation for energy-efficient fog networks includes costs only in selected parts of these networks. In [6,7,8,9,10] computational requests can be distributed between various combinations of MDs, one or more nearby FNs and a remote cloud. These studies optimize energy consumption either alone [7,10] or in addition to other parameters [6,8,9]. However, what differentiates our work from those is that they only consider energy consumption from the perspective of MDs. In contrast, we look at the total energy spent on computation as well as wireless and wired transmission in the entire network.

Other studies, similarly to ours, examine energy consumption within the fog network but ignore, e.g., costs related to transmission between different FNs (FN-FN) [11,12,13], transmission between MDs and FNs (MD-FN) [11,12,13] or transmission between FNs and the cloud (FN-CN) [13,14]. In some studies, the possibility of FN-FN [14,15] and FN-CN [15] is not considered at all. In [11,12,13,14,16], computational requests are not examined individually, but as aggregated data. In our work, each request is characterized by its own set of parameters such as size, computational complexity and delay requirement. Moreover, no optimization problem related to processing requests is proposed in [12,16].

A summary of related articles in contrast to our work is presented in Table 1. Rows MD-FN, FN-FN and FN-CN represent costs related to transmission between nodes while rows MD, FN and CN represent costs related to computations at given nodes. The notation used is as follows: Optim. means that energy and delay are optimized, Cons. means that these are considered in calculations and Ign. means that these are ignored or assumed negligible. N/A means that in a network modeled in a given work, there is no possibility of such transmission/computation—energy and delay costs are not applicable. E stands for energy and D stands for delay. If both are considered/optimized/ignored this notation is skipped. Sets means the allocation of sets of individual requests (characterized by size, required computations, etc.) is considered. Flow, on the other hand, means that the requests are not considered individually but as a total bitrate, rate of computations, etc., that have to be completed.

This work extends [17] with the following novel aspects: (i) optimizing the wireless connection of end devices to the fog tier; (ii) introducing an additional set of transmission allocation variables to the optimization problem and its solution; (iii) providing an analytical solution to the proposed problem; (iv) examining the effectiveness of new heuristic algorithms with constraints on either wired or wireless transmission.

### 1.3. Contribution and Work Outline

The main contribution of this work is a complete analysis of the energy required to satisfy a computation request. A sophisticated nonlinear optimization problem is formulated with the objective of minimizing the energy consumed for the computation and transportation of tasks under delay constraints. We propose a solution by dividing the problem into subproblems where optimal values of CPU frequencies, transmission paths and allocations of computational tasks to nodes are found. Unlike similar works which depend on various heuristics, we propose an analytical approach that guarantees that we find the optimal solution.

This work is structured as follows. The network model is presented in Section 2. The optimization problem is formulated in Section 3, while its solution is proposed in Section 4. Section 5 contains simulation results and Section 6 presents the conclusion.

## 2. Network Model

Let us present the three-tier network model used in this work. In the bottom tier of the network, there is a set M of MD (e.g., smartphones) with specific computational requests. We assume that serving these tasks requires offloading them to one of the FN or CN, constituting the second and the third tier, respectively. The MDs cannot process these tasks on their own because of energy or computational limitations. The MDs send computational requests using wireless transmission to one of the nearby FNs. As shown in Figure 1, FNs are located at BS or AP, close to the end users. Then, each task can be processed either in one of the FNs out of set F or in the cloud tier (set C of CN). Unlike MD, nodes in the fog and cloud tiers of the network are interconnected with wire-based communication technology.

The model shown in this work extends the one used in [17]. The notation used for modelling the network is shown in Table 2.

### 2.1. Computational Requests

Let T be a numbered set $\left\{T_{1},T_{2},...,T_{\left|T\right|}\right\}$ of all time instances at which MDs offload computational requests. Let $R_{k}$ be a set of all requests that MDs try to offload at time $T_{k}$. The following parameters characterize each computing request $r\in R_{k}$:

- MD $m^{r}\in M$, which offloads the task (letters in superscript are used throughout this work as upper indices, nor exponents, e.g., $m^{r}$ does **not** denote *m* to the power of *r*);
- Size $L^{r}$ in bits;
- Arithmetic intensity $\theta^{r}$ in FLOP/bit;
- Ratio $o^{r}$ of the size of the result of the processed task *r* to the size of the offloaded task *r*;
- Maximum tolerated delay $D_{\max}^{r}$.

Let us define a binary variable $a_{n}^{r}$ that shows where the request is computed, i.e., $a_{n}^{r}$ equals 1 if $r\in R_{k}$ is computed at node n∈F∪C and 0 otherwise. Similarly, let us define a binary variable $w_{l}^{r}$ that indicates if request *r* is wirelessly transmitted from MD $m^{r}$ to FN n∈F.

**Table 2:** The notation used for modeling the network and defining the optimization problem.

Symbol	Description
T	set $\left\{T_{1},\cdots ,T_{\left|T\right|}\right\}$ of all considered time instances, when one or more computational request arrives
M	set of all Mobile/End Devices
F	set of all Fog Nodes
C	set of all Cloud Nodes
$R_{k}$	set of all computational requests arriving at $T_{k}\in T$
$L^{r}$	size of request $r\in R_{k}$
$\theta^{r}$	computational complexity of request $r\in R_{k}$
$m^{r}$	MD which offloads the request $r\in R_{k}$
$o^{r}$	output-to-input data size ratio of request $r\in R_{k}$
$D_{\max}^{r}$	maximum tolerated delay requirement for request $r\in R_{k}$
$T_{k}$	time at which request $r\in R_{k}$ arrives in the network, k∈{1,⋯,|T|}
$\gamma_{y}^{x}$	energy-per-bit cost of transmitting data between nodes *x* and *y*
$s_{n}$	number of FLOPs performed per single clock cycle at node n∈N
$b_{y}^{x}$	link bitrate between nodes *x* and *y*
$d^{n}$	fiberline distance to CN n∈C
χ	a parameter characterizing delay depending on distance
$f_{\min,n}$	minimum clock frequency of node n∈N
$f_{\max,n}$	maximum clock frequency of node n∈N
$p_{n,0}$, $p_{n,1}$, $p_{n,2}$, $p_{n,3}$	parameters of the power model of CPU installed in node n∈N
$t_{n}$	time at which node n∈N finishes computing its last task
$a_{n}^{r}$	variable showing whether request $r\in R_{k}$ is computed at node n∈N, $a_{n}^{r}\in \left\{0,1\right\}$
$w_{l}^{r}$	variable showing whether request $r\in R_{k}$ is transmitted wirelessly to node l∈F, $w_{l}^{r}\in \left\{0,1\right\}$
$f_{n}$	clock frequency of node n∈N, $f_{\min,n}\leq f_{n}\leq f_{\max,n}$
$\beta_{n}$	energy efficiency (FLOPS per Watt) characterizing node n∈N
$P_{n}$	power consumption related to computations at node n∈N
$E_{\mathrm{tot}}^{r}$	energy spent on transmission and processing of request $r\in R_{k}$
$E_{\mathrm{cp}}^{r}$	energy spent in the network on processing request $r\in R_{k}$
$E_{\mathrm{comm}}^{r}$	energy spent on transmission of request $r\in R_{k}$
$E_{\mathrm{wl}}^{r}$, $E_{\mathrm{wd}}^{r}$	energy spent on wireless/wired transmission of request $r\in R_{k}$
$E_{\mathrm{comm},y}^{r,x}$	energy cost for transmission of request $r\in R_{k}$ between nodes *x* and *y*
$E_{\mathrm{cp},n}^{r}$	energy cost of processing request $r\in R_{k}$ at node n∈N
$D{}_{\mathrm{tot}}^{r}$	total delay of request $r\in R_{k}$
$D_{\mathrm{comm}}^{r}$	delay caused by transmitting request $r\in R_{k}$
$D_{\mathrm{wl}}^{r}$, $D_{\mathrm{wd}}^{r}$	wireless/wired delay of request $r\in R_{k}$
$D_{\mathrm{comm},y}^{r,x}$	delay of transmission of request $r\in R_{k}$ between nodes *x* and *y*
$D_{\mathrm{ul},n}^{r,m^{r},l}$	uplink delay of transmitting request $r\in R_{k}$ to node n∈F, provided that $w_{l}^{r}=1$
$D_{\mathrm{queue}}^{r}$	queuing delay of request $r\in R_{k}$
$D_{\mathrm{queue},n}^{r,l}$	queuing delay of request $r\in R_{k}$ at node n∈N, provided that $w_{l}^{r}=1$
$D_{\mathrm{cp}}^{r}$	computational delay caused by processing request $r\in R_{k}$
$D_{\mathrm{cp},n}^{r}$	computational delay caused by processing request $r\in R_{k}$ at node n∈N

### 2.2. Energy Consumption

The energy consumption model is divided into two parts: computation (processing of data) and communication (transmission of data). Energy $E_{\mathrm{cp}}^{r}$ spent on computing request $r\in R_{k}$ equals:

(1) $$E_{\mathrm{cp}}^{r}=\sum_{n\in F\cup C}a_{n}^{r}E_{\mathrm{cp},n}^{r}=\sum_{n\in F\cup C}a_{n}^{r}\frac{L^{r}\theta^{r}}{\beta_{n}},$$

where $E_{\mathrm{cp},n}^{r}$ is the energy spent on computing request $r\in R_{k}$ at node n∈F∪C and $\beta_{n}$ is the computational efficiency of node n∈F∪C given in FLOPS per watt [18]. For CN, we assume constant CPU clock frequency $f_{n}$ and efficiency $\beta_{n}$. For FN, $\beta_{n}$ depends on CPU frequency $f_{n}$ of node n∈F, number $s_{n}$ of FLOP performed within a single clock cycle of CPU [19] and on power consumption $P_{n}$ of CPU. $\beta_{n}$ is obtained by modeling $P_{n}$ as a polynomial function of $f_{n}$ using four parameters $p_{n,3}$, $p_{n,2}$, $p_{n,1}$ and $p_{n,0}$ derived from [20]:

(2) $$\beta_{n}=\frac{f_{n}s_{n}}{P_{n}}=\frac{f_{n}s_{n}}{p_{n,3}f_{n}^{3}+p_{n,2}f_{n}^{2}+p_{n,1}f_{n}+p_{n,0}}.$$

This representation provides the flexibility to cover various models of CPU. The clock frequency $f_{n}$ must lie within the range of possible frequencies of CPU in node n∈F, i.e., $f_{\min,n}\leq f_{n}\leq f_{\max,n}$.

The energy spent on the transmission of request $r\in R_{k}$ is the sum of energies resulting from wireless ($E_{\mathrm{wl}}^{r}$) and wired ($E_{\mathrm{wd}}^{r}$) communication:

(3) $$E_{\mathrm{comm}}^{r}=E_{\mathrm{wl}}^{r}+E_{\mathrm{wd}}^{r}.$$

The energy spent on wireless transmission of request $r\in R_{k}$ equals:

(4) $$E_{\mathrm{wl}}^{r}=\sum_{l\in F}w_{l}^{r}E_{\mathrm{comm},l}^{r,m^{r}}=\sum_{l\in F}w_{l}^{r}L^{r}\left(1+o^{r}\right)\gamma_{l}^{m^{r}},$$

where $E_{\mathrm{comm},l}^{r,m^{r}}$ is the energy required to transmit request $r\in R_{k}$ from MD $m^{r}\in M$ to FN l∈F and return the calculation result in the reverse direction, while $\gamma_{l}^{m^{r}}$ is the energy-per-bit cost of this transmission. $L^{r}o^{r}$ is the size (in bits) of results transmitted back to MD $m^{r}$.

The energy spent on wired transmission of request $r\in R_{k}$ equals:

(5) $$E_{\mathrm{wd}}^{r}=\sum_{l\in F}w_{l}^{r}\sum_{n\in F\cup C}a_{n}^{r}E_{\mathrm{comm},n}^{r,l}=\sum_{l\in F}w_{l}^{r}\sum_{n\in F\cup C}a_{n}^{r}L^{r}\left(1+o^{r}\right)\gamma_{n}^{l},$$

where $E_{\mathrm{comm},n}^{r,l}$ is the energy required to transmit request $r\in R_{k}$ between FN l∈F and node n∈F∪C, while $\gamma_{n}^{l}$ is the energy-per-bit cost of this transmission. Energy-per-bit cost can be derived from [21], where the power consumption of networking equipment increases linearly with load starting from idle power. This relation can also be seen in measurements of core routers [22,23]. There is no wired communication between nodes if the request is processed at the same node to which it is wirelessly transmitted by the MD, i.e., $\forall l\in F\;\gamma_{l}^{l}=0$.

The total energy spent on offloading request $r\in R_{k}$ is given by:

(6) $$E_{\mathrm{tot}}^{r}=E_{\mathrm{cp}}^{r}+E_{\mathrm{wl}}^{r}+E_{\mathrm{wd}}^{r}.$$

### 2.3. Delay

Three components form the delay model: communication, processing and queuing. The delay $D_{\mathrm{cp}}^{r}$ caused by computing request $r\in R_{k}$ equals:

(7) $$D_{\mathrm{cp}}^{r}=\sum_{n\in F\cup C}a_{n}^{r}D_{\mathrm{cp},n}^{r}=\sum_{n\in F\cup C}a_{n}^{r}\frac{L^{r}\theta^{r}}{f_{n}s_{n}},$$

where $D_{\mathrm{cp},n}^{r}$ is the time required to compute request $r\in R_{k}$ at node n∈F∪C.

The delay caused by communication can be further subdivided into wireless ($D_{\mathrm{wl}}^{r}$) and wired ($D_{\mathrm{wd}}^{r}$) delay:

(8) $$D_{\mathrm{comm}}^{r}=D_{\mathrm{wl}}^{r}+D_{\mathrm{wd}}^{r}.$$

The delay caused by wireless transmission of request $r\in R_{k}$ equals:

(9) $$D_{\mathrm{wl}}^{r}=\sum_{l\in F}w_{l}^{r}D_{\mathrm{comm},l}^{r,m^{r}}=\sum_{l\in F}w_{l}^{r}\frac{L^{r}\left(1+o^{r}\right)}{b_{l}^{m^{r}}},$$

where $D_{\mathrm{comm},l}^{r,m^{r}}$ is the time required to transmit request $r\in R_{k}$ between MD $m^{r}\in M$ and FN l∈F, while $b_{l}^{m^{r}}$ is the bitrate of this transmission between FN *l* and MD $m^{r}$.

The delay caused by wired transmission of request $r\in R_{k}$ equals:

(10) $$D_{\mathrm{wd}}^{r}=\sum_{l\in F}w_{l}^{r}\sum_{n\in F\cup C}a_{n}^{r}D_{\mathrm{comm},n}^{r,l},$$

where $D_{\mathrm{comm},n}^{r,l}$ is the time required to transmit request $r\in R_{k}$ between FN l∈F and node n∈F∪C. The model for calculation of $D_{\mathrm{comm},n}^{r,l}$ differs depending on whether node *n* is an FN or a CN. It is assumed that cloud data centers are located away from the rest of the network (hundreds or even thousands of kilometers away) which requires the distance-related delay to be modeled. The delay caused by transmitting request $r\in R_{k}$ between (to and from) FN l∈F and cloud node n∈C is:

(11) $$D_{\mathrm{comm},n}^{r,l}=\frac{L^{r}\left(1+o^{r}\right)}{b_{n}^{l}}+d^{n}\cdot \chi ,$$

where $b_{n}^{l}$ is the link bitrate in the backhaul and backbone network between nodes *l* and *n*, while $d^{n}$ is the fiberline distance to CN n∈C. The parameter χ indicates the rate at which delay increases with distance $d^{n}$ [24].

For transmission between FNs, we assume the delay caused by the distance between them ($d^{n}\cdot \chi$ in Equation (11)) is negligible—well below 1 ms as we use a value of 7.5μs/km for parameter χ [24]—and therefore we ignore it. Delay caused by communication between FN l∈F and n∈F for request $r\in R_{k}$ equals:

(12) $$D_{\mathrm{comm},n}^{r,l}=\frac{L^{r}\left(1+o^{r}\right)}{b_{n}^{l}}.$$

The special case is when the request *r* is received wirelessly at FN *n* and the same node is used for processing. In this case, no wired communication delay is expected, i.e., $D_{\mathrm{comm},n}^{r,n}=0,\forall n\in F$.

Even more significant differences can be observed while modeling queuing delays for requests processed in the fog tier and in the cloud tier of the network. This stems from the fact that clouds are assumed to have huge (practically infinite) computational resources with parallel-computing capabilities and there is no need to queue multiple requests served by the CN n∈C. They can be processed simultaneously. Meanwhile, if multiple requests are sent to the same FN n∈F for processing in a short time span, additional delays may occur due to congestion of computational requests (an arriving request cannot be processed until processing of all the previous requests has been completed). We define a scheduling variable $t_{n}\in R^{+}$ to represent the point in time at which the last request scheduled at FN n∈F is finished processing. The queuing delay of request $r\in R_{k}$, transmitted wirelessly to node l∈F, for computations being carried at node n∈F equals:

(13) $$D_{\mathrm{queue},n}^{r,l}=\max\left(0,t_{n}-T_{k}-D_{\mathrm{ul},n}^{r,m^{r},l}\right),$$

where $D_{\mathrm{ul},n}^{r,m^{r},l}=\frac{1}{1+o^{r}}\left(D_{\mathrm{comm},l}^{r,m^{r}}+D_{\mathrm{comm},n}^{r,l}\right)$ is the uplink delay of transmitting request *r* to node *n* through FN *l*. $D_{\mathrm{queue},n}^{r,l}$ has nonzero values when $t_{n}>T_{k}+D_{\mathrm{ul},n}^{r,m^{r},l}$. In such cases, the request *r* arrives at node *n* at time $T_{k}+D_{\mathrm{ul},n}^{r,m^{r},l}$. It is kept in a queue until time $t_{n}$, when processing of another request (or requests) ends. For each node n∈C, $D_{\mathrm{queue},n}^{r,l}$ always equals zero—due to the parallel processing powers of the cloud, each request may be computed right away, regardless of how many requests are already being processed. Queuing delay of request $r\in R_{k}$ is:

(14) $$D_{\mathrm{queue}}^{r}=\sum_{l\in F}w_{l}^{r}\sum_{n\in F\cup C}a_{n}^{r}D_{\mathrm{queue},n}^{r,l}.$$

Finally, the total delay of processing request $r\in R_{k}$ equals the sum of delays related to computation, transmission and queuing:

(15) $$D{}_{\mathrm{tot}}^{r}=D_{\mathrm{cp}}^{r}+D_{\mathrm{comm}}^{r}+D_{\mathrm{queue}}^{r}.$$

### 2.4. Updating Scheduling Variables in the Fog

Since no requests are processed when a simulation starts, we set $t_{n}=0,\forall n\in F.$ Then, for each $T_{k}\in T$, after allocations $a_{n}^{r}$ and $w_{l}^{r}$ are determined, the times $t_{n}$ are updated for every n∈F according to when computation of requests offloaded to the FN are scheduled to finish:


(16) $$t_{n}:=\max\left(t_{n},T_{k}+\sum_{r\in R_{k}}\sum_{l\in F}a_{n}^{r}w_{l}^{r}\left(D_{\mathrm{ul},n}^{r,m^{r},l}+D_{\mathrm{queue},n}^{r,l}+D_{\mathrm{cp},n}^{r}\right)\right).$$

## 3. Optimization Problem

Our defined problem seeks to minimize the total energy cost of offloading all requests that enter the network at time $T_{k}$, that is to find:

(17) $$\left(a^{☆},w^{☆},f^{☆}\right)=\arg\min_{a,f,w}\sum_{r\in R}E_{\mathrm{tot}}^{r},$$

subject to:

(18) $$\begin{array}{cccc} \; & \;\sum_{n\in F\cup C}a_{n}^{r}=1 & \; & \forall r\in R_{k}, \end{array}$$

(19) $$\begin{array}{cccc} \; & \;\sum_{r\in R_{k}}a_{n}^{r}\leq 1, & \; & \forall n\in F, \end{array}$$

(20) $$\begin{array}{cccc} \; & \;\sum_{l\in F}w_{l}^{r}=1 & \; & \forall r\in R_{k}, \end{array}$$

(21) $$\begin{array}{cccc} \; & \;D{}_{\mathrm{tot}}^{r}\leq D_{\max}^{r}, & \; & \forall r\in R_{k}, \end{array}$$

(22) $$\begin{array}{cccc} \; & \;f_{\min,n}\leq f_{n}\leq f_{\max,n},\;f_{n}\in R & \; & \forall n\in F, \end{array}$$

(23) $$\begin{array}{cccc} \; & \;a_{n}^{r}\in \left\{0,1\right\}, & \; & \forall r\in R_{k},\;\forall n\in F\cup C, \end{array}$$

(24) $$\begin{array}{cccc} \; & \;w_{l}^{r}\in \left\{0,1\right\}, & \; & \forall r\in R_{k},\;\forall l\in F, \end{array}$$

where $a^{☆}=\left\{a{{}_{n}^{r}}^{☆}\right\}$, $w^{☆}=\left\{w{{}_{l}^{r}}^{☆}\right\}$ and $f^{☆}=\left\{f_{n}^{☆}\right\}$ are the optimal values of allocation variables $a_{n}^{r}$ and $w_{l}^{r}$ and CPU clock frequencies $f_{n}$, respectively. Constraints (18) guarantee that each request must be processed at exactly one FN or CN. Constraints (19) stipulate that no more than a single request can be processed at a given FN at a given time. Constraints (20) guarantee that for each request, a single FN will be used for wireless connectivity. Constraints (21) guarantee that the total delay must not be greater than the maximum acceptable one. Constraints (22) show the lower and upper bounds of CPU frequency at each FN. Finally, according to Constraints (23) and (24), decision variables $a_{n}^{r}$ and $w_{l}^{r}$ take only binary values.

There exist sets of requests $R_{k}$ for which the optimization cannot be solved (e.g., there is no feasible allocation of requests so that each request is processed (18) while fulfilling its delay requirement (21)). In such a scenario, we decide to reject requests for which (21) cannot be satisfied rather than ending the optimization without finding a solution (which would translate into rejecting all requests $R_{k}$). The remaining requests (set $R_{k}\setminus R_{k}^{\prime }$, where $R_{k}^{\prime }$ denotes the set of rejected requests) are then subjected to the optimization.

## 4. Problem Solution

In this section we provide a step-by-step solution to the optimization problem. In short, we first find minimum operating frequencies at which delay requirements of offloaded requests are met. Then, we find optimal operating frequencies which minimize energy consumption spent on computations for given combinations of nodes and requests. At this point combinations which cannot satisfy delay requirements are known. Then, the nodes to which wireless transmission energy costs are the lowest are found. Finally, we assign requests to nodes for computing to minimize the total energy consumption. This linear assignment problem is solved with the Hungarian algorithm [25,26]. Notation used in our solution is summarized in Table 3.

### 4.1. Auxiliary Variables

Let us define the auxiliary variable $f_{n,l}^{r}$ as the CPU frequency of node n∈F∪C where request $r\in R_{k}$ is allocated while node l∈F is the node to which *r* is wirelessly transmitted ($w_{l}^{r}$ = 1). The relation between $f_{n,l}^{r}$ and $f_{n}$ is given by $f_{n}=\sum_{r\in R_{k}}\sum_{l\in F}a_{n}^{r}w_{l,n}^{r}f_{n,l}^{r}$. Similarly, $w_{l,n}^{r}$ determines which node l∈F request $r\in R_{k}$ is wirelessly transmitted to provided that it is allocated to n∈F∪C ($a_{n}^{r}$ = 1) and $w_{l}^{r}=\sum_{n\in F\cup C}w_{l,n}^{r}$. Moreover, let $D{}_{\mathrm{tot},n}^{r,l}$ be the total delay of request $r\in R_{k}$ provided that it is computed at node n∈F∪C ($a_{n}^{r}$ = 1) and node l∈F be the node to which *r* is wirelessly transmitted ($w_{l}^{r}$ = 1).

**Table 3:** Additional notation used in the problem solution.

Symbol	Description
$w_{l,n}^{r}$	variable showing whether request $r\in R_{k}$ is transmitted wirelessly to node l∈F, provided that $a_{n}^{r}=1$
$f_{n,l}^{r}$	clock frequency of node n∈N, provided that $a_{n}^{r}=1$ and $w_{l}^{r}=1$, $r\in R_{k}$
$D{}_{\mathrm{tot},n}^{r,l}$	total delay of $r\in R_{k}$, provided that $a_{n}^{r}=1$ and $w_{l}^{r}=1$
$D{}_{\mathrm{cp},n}^{r,l}$	computational delay of $r\in R_{k}$, provided that $a_{n}^{r}=1$ and $w_{l}^{r}=1$
$E_{\mathrm{cp},n,l}^{r}$	energy spent on processing of request $r\in R_{k}$, provided that $a_{n}^{r}=1$ and $w_{l}^{r}=1$
$R_{k}^{\prime }$	set of requests rejected due to delay requirements
${\hat{R}}_{k}$	set of not rejected requests, ${\hat{R}}_{k}=R_{k}\backslash R_{k}^{\prime }$
$w_{l,n}^{r}$	variable showing whether request $r\in R_{k}$ is transmitted wirelessly to node l∈F, provided that $a_{n}^{r}=1$
$f_{n,l}^{r}$	clock frequency of node n∈N, provided that $a_{n}^{r}=1$ and $w_{l}^{r}=1$, $r\in R_{k}$
$D{}_{\mathrm{tot},n}^{r,l}$	total delay of $r\in R_{k}$, provided that $a_{n}^{r}=1$ and $w_{l}^{r}=1$
$D{}_{\mathrm{cp},n}^{r,l}$	computational delay of $r\in R_{k}$, provided that $a_{n}^{r}=1$ and $w_{l}^{r}=1$
$E_{\mathrm{cp},n,l}^{r}$	energy spent on processing of request $r\in R_{k}$, provided that $a_{n}^{r}=1$ and $w_{l}^{r}=1$
$R_{k}^{\prime }$	set of requests rejected due to delay requirements
${\hat{R}}_{k}$	set of not rejected requests, ${\hat{R}}_{k}=R_{k}\backslash R_{k}^{\prime }$

### 4.2. Finding Optimal Frequencies

Let us rewrite (19) by expanding $E_{\mathrm{tot}}^{r}$ into parts caused by computations ($E_{\mathrm{cp},n}^{r}$), wireless transmission ($E_{\mathrm{comm},l}^{r,m^{r}}$, between MD $m^{r}$ and node *l*) and wired transmission ($E_{\mathrm{comm},n}^{r,l}$, between nodes *l* and *n*):


(25) $$\left(a^{☆},w^{☆},f^{☆}\right)=\arg\min_{a,w,f}\sum_{r\in R_{k}}\sum_{l\in F}\sum_{n\in F\cup C}a_{n}^{r}w_{l}^{r}\left(E_{\mathrm{cp},n}^{r}+E_{\mathrm{comm},l}^{r,m^{r}}+E_{\mathrm{comm},n}^{r,l}\right).$$

Out of these three parts, $E_{\mathrm{cp},n}^{r}$ is the only one that depends on frequencies $f_{n}$. The goal of this step is to find $f_{n,l}^{r}{}^{☆}$, i.e., values of $f_{n}$ which minimize $E_{\mathrm{cp},n}^{r}$ for all possible values of $a_{n}^{r}$ and $w_{l}^{r}$. The only constraints that depend on values of $f_{n,l}^{r}$ are (21) and (22).

The minimum values of $f_{n,l}^{r}$ which satisfy Constraints (21) can be obtained by solving the inequality $D{}_{\mathrm{tot},n}^{r,l}\leq D_{\max}^{r}$.

(26) $$D{}_{\mathrm{cp},n}^{r,l}+D_{\mathrm{comm},l}^{r,m^{r}}+D_{\mathrm{comm},n}^{r,l}+D_{\mathrm{queue},n}^{r,l}\leq D_{\max}^{r}$$

(27) $$\frac{L^{r}\theta^{r}}{s_{n}f_{n,l}^{r}}+D_{\mathrm{comm},l}^{r,m^{r}}+D_{\mathrm{comm},n}^{r,l}+D_{\mathrm{queue},n}^{r,l}\leq D_{\max}^{r}$$

(28) $$f_{n,l}^{r}\geq \frac{L^{r}\theta^{r}}{s_{n}\left(D_{\max}^{r}-D_{\mathrm{comm},l}^{r,m^{r}}-D_{\mathrm{comm},n}^{r,l}-D_{\mathrm{queue},n}^{r,l}\right)}≜f_{\min,n,l}^{r}$$

Let us rewrite $E_{\mathrm{cp},n}^{r}$ as a function of $f_{n}$ based on (1) and (2).

(29) $$E_{\mathrm{cp},n}^{r}\left(f_{n}\right)=\frac{L^{r}\theta^{r}\left(p_{n,3}f_{n}^{3}+p_{n,2}f_{n}^{2}+p_{n,1}f_{n}+p_{n,0}\right)}{f_{n}s_{n}}$$

Its derivative with respect to $f_{n}$ equals:

(30) $$E_{\mathrm{cp},n}^{r}{}^{\prime }\left(f_{n}\right)=\frac{L^{r}\theta^{r}}{s_{n}}\frac{\left(2p_{n,3}f_{n}^{3}+p_{n,2}f_{n}^{2}-p_{n,0}\right)}{f_{n}^{2}}.$$

The function $E_{\mathrm{cp},n}^{r}\left(f_{n}\right)$ is continuous and differentiable for positive $f_{n}$ (the only discontinuity is at $f_{n}=0$). Therefore, its extrema in a given interval can only be found at the bounds of this interval or for points at which the derivative equals zero. $E_{\mathrm{cp},n}^{r}{}^{\prime }\left(f_{n}\right)$ has a cubic function in the numerator, so it has at most three real roots.

Now, we find $f_{n,l}^{r}{}^{☆}$ for $r\in R_{k}$, n∈F, w∈F by finding the minimum of $E_{\mathrm{cp},n}^{r}\left(f_{n}\right)$ in the interval $\left[\max\left(f_{\min,n,l}^{r},f_{\min,n}\right),f_{\max,n}\right]$. The corresponding minimum energy costs are as follows:

(31) $$E_{\mathrm{cp},n,l}^{r}{}^{☆}=E_{\mathrm{cp},n}^{r}\left(f_{n,l}^{r}{}^{☆}\right).$$

For values $r\in R_{k}$, n∈F, w∈F for which $f_{\min,n,l}^{r}>f_{\max,n}$, constraints (21) and (22) cannot both be satisfied, so we set $E_{\mathrm{cp},n,l}^{r}{}^{☆}$ to infinity. For computations in clouds n∈C, we do not optimize the frequency $f_{n}$ ($f_{n}=\mathrm{const}.$, $E_{\mathrm{cp},n,l}^{r}{}^{☆}=E_{\mathrm{cp},n}^{r}\left(f_{n}\right)$). For values $r\in R_{k}$, n∈C, w∈F for which $f_{\min,n,l}^{r}>f_{n}$, constraint (21) cannot be satisfied, i.e., we set $E_{\mathrm{cp},n,l}^{r}{}^{☆}$ to infinity.

Each request $r\in R_{k}$ for which the following occurs:

(32) $$E_{\mathrm{cp},n,l}^{r}{}^{☆}=\infty ,\forall n\forall l$$

cannot be fully processed within their delay requirements regardless of chosen computation/transmission nodes. All such requests are therefore rejected. The remaining optimization is performed over ${\hat{R}}_{k}=R_{k}\backslash R_{k}^{\prime }$, where $R_{k}^{\prime }$ is the set of rejected requests.

### 4.3. Transmission Allocation

The auxiliary matrix $w_{n}^{☆}=\left\{w_{l,n}^{r}{}^{☆}\right\}$ can be obtained. For each task $r\in R_{k}$ and each computing node n∈F∪C, the goal is to choose node l∈F, which minimizes the sum of energy spent on computations (calculated and optimized in the previous step) and transmission (depending directly on $w_{l,n}^{r}$), i.e., to find:

(33) $$w_{n}^{☆}=\arg\min_{w_{n}}\sum_{l\in F}w_{l,n}^{r}\left(E_{\mathrm{cp},n,l}^{r}{}^{☆}+E_{\mathrm{comm},l}^{r,m^{r}}+E_{\mathrm{comm},n}^{r,l}\right),$$

while satisfying (20) and (24). This is equivalent to finding nodes *l*, which minimize the expression $\left(E_{\mathrm{cp},n,l}^{r}{}^{☆}+E_{\mathrm{comm},l}^{r,m^{r}}+E_{\mathrm{comm},n}^{r,l}\right)$.

### 4.4. Computation Allocation

The vector $a^{☆}$ can now be obtained by solving the simplified problem:

(34) $$a^{☆}=\arg\min_{a}\sum_{r\in R_{k}}\sum_{l\in F}\sum_{n\in F\cup C}a_{n}^{r}{w_{l,n}^{r}}^{☆}\left(E_{\mathrm{cp},n,l}^{r}{}^{☆}+E_{\mathrm{comm},l}^{r,m^{r}}+E_{\mathrm{comm},n}^{r,l}\right),$$

subject to (18), (19) and (23). This corresponds to the linear assignment problem [25]—each request $r\in {\hat{R}}_{k}$ is assigned to one and only one node n∈F∪C. The cost matrix has $\left|{\hat{R}}_{k}\right|$ rows and $\left|F\right|+\left|{\hat{R}}_{k}\right|\cdot \left|C\right|$ columns. The columns representing processing at FN are used once as each of them can serve one request at a time while the columns representing processing at CN are multiplied to ensure that multiple requests can be assigned to them simultaneously. The Hungarian algorithm [25,26] is used to solve this problem.

## 5. Results

Results obtained from computer (MATLAB) simulations and their setup are presented in this section. While the main goal is to serve all the incoming requests within allowed latency constraints with minimum energy, requests that failed to be served are set with virtually infinite consumed energy. This facilitates a fair comparison of various request allocation strategies using only distribution of energy consumption spent per offloaded request. Therefore, we choose medians, percentiles and CDF as evaluation metrics.

For the purpose of computing medians and other percentiles in this section, the energy costs related to rejected requests are equal to positive infinity—such an approach (as well as using other fixed values or omitting them entirely) has a considerably larger impact on the averages. Medians and percentiles avoid bias that unserved requests have with respect to average values.

### 5.1. Scenario Overview

**Figure 2:**
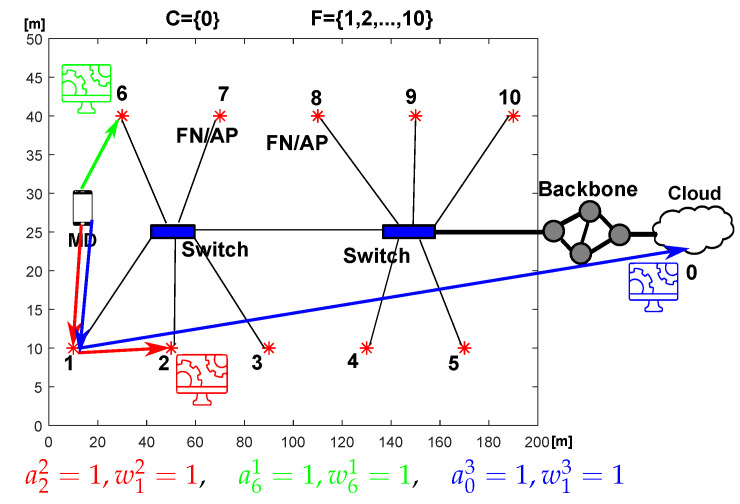
Diagram of the considered network composed of 10 FNs and a cloud with three examples of request allocations.

Let us consider a network with |F|=10 FN and |C|=1 cloud DC. Simulation parameters are summarized in Table 4. Figure 2 shows a connection diagram between these FNs and the cloud. The examined environment represents a commercial facility such as an airport, where the end users (MD) want to have their requests processed. Moreover, Figure 2 presents three examples of requests being calculated: (i) in the same FN as the utilized AP, (ii) being calculated in another FN and (iii) being offloaded to the cloud. Appropriate values of binary variables $a_{n}^{r}$ and $w_{l}^{r}$ are presented in Figure 2.

*Requests*—between 5 and 10 new computational requests with uniform distribution at time $T_{k}\in T$ appear. These requests appear at random locations within the area of the examined network (with uniform distribution in both dimensions). The value $T_{k}$ is generated as a random delay after the previous time instance $T_{k-1}$. The difference $T_{k}-T_{k-1}$ is chosen to be a random variable of exponential distribution with an average value of 200ms. The requests have randomly assigned values of their parameters (size, arithmetic intensity, delay requirement) in ranges shown in Table 4 with uniform distribution.

*Computations*—each FN has computational resources and a frequency–power relationship of a single Intel Core i5-2500K as its CPU. Data relating frequency, voltage and power consumption of i5-2500K are taken from [27] and inserted into Equation (2) adapted from [20] to obtain values for $p_{n,3}$, $p_{n,2}$, $p_{n,1}$ and $p_{n,0}$. The parameter *s* equals 16 for this CPU [19]. The resulting computational efficiency β is the highest (0.9586 GFLOPS/W) at frequency f=2.6063 GHz.

To simulate a scenario with varying computational efficiencies of nodes, we multiply the resulting computational efficiency (2) by random values from the range [0.5,1.5] generated independently for each node n∈F.

As for the computational capability of the cloud, its CPUs are parameterized according to the *Intel Xeon Phi* family commonly used in computer clusters [18,32] run at constant frequency f=1.5 GHz characterized with s=32 [19].

*Wireless transmission*—the power consumption model of the wireless transmission is based on [29] and depends on the data rate and path loss. We use values derived for ASUS USB-N10 WiFi card. The path loss values are determined using the model from Section 3.1 of [31] for a commercial area and frequency closest to 2.4GHz (20 dB for frequency 2.1GHz). The wireless link uses a maximum available rate that depends on the minimum sensitivity specified in Section 19.3.19.2 of [30] for a given modulation and coding scheme. It ranges from 6.5 Mbps (BPSK, 1/2) at −82 dBm to 65 Mbps (64-QAM, 5/6) at −64 dBm. The energy-per-bit cost $\gamma_{l}^{m^{r}}$ is obtained by dividing the power by the wireless link data rate.

*Wired transmission*—in order to derive energy-per-bit cost of transmitting requests from one node to another (i.e., $\gamma_{n}^{l}$ from l∈F to n∈F∪C), we need to add costs induced in all devices through which it flows. For the power consumption of a single networking device, the linear model from [21] is used. It includes idle power $P_{idle}$ and active power that scales with load *C* (in bits/second) by parameter γ (in Joules/bit):

(35) $$P=P_{idle}+C\frac{P_{max}-P_{idle}}{C_{max}}=P_{idle}+C\gamma ,$$

where $P_{max}$ denotes maximum power consumption and $C_{max}$ denotes maximum load. Energy-per-bit cost of transmitting data $\gamma_{n}^{l}$ is equal to the sum of γ parameters of all network devices through which the data flows between nodes *l* and *n*. In this work, we assume $\gamma_{n}^{l}=\gamma_{l}^{n}$. It is assumed for the connections between FN that they are connected with 1 G Ethernet. The power consumption of Ethernet switches is set according to [28,33]. Each switch can serve up to 6 FN on the LAN side with 1 Gbps links (star topology) and can be connected to the 10 G EPON on the WAN side. Cost-per-bit of transmission through these switches is equal to 2 nJ/bit (82 W at 1 Gbps throughput, 80 W with no traffic). The configuration can be seen in Figure 2 showing 10 FNs connected with 2 switches.

For the connection between the fog tier of the network and the cloud, it is assumed that the data flow through multiple nodes. Olbrich et al. [24] use geographically locatable nodes (over 250 nodes around the globe) to derive multiple path characteristics. Their results show that the RTT of a packet is, on average, 1.5 times longer than an estimation based only on fiberline distance (the speed of light in optic fiber $\approx 2\times 10^{8}$ m/s, in vacuum $c\approx 3\times 10^{8}$ m/s). The measured RTT has a slope of 7.5μ s/km. We assign this 7.5μ s/km value to parameter χ. The Cloud DC is assumed to be located 2000 km away from the rest of the network. It is estimated that the energy-per-bit cost of transmitting data through the backbone network to the Cloud is equal to 12.66 nJ/bit based on 12 Juniper T1600 routers—each with cost-per-bit equal 1.03 nJ/bit [12,22] and a 10G EPON gateway with 0.3 nJ/bit cost [34]. While there is other equipment through which the data flow within the core network (e.g., optical amplifiers), the value 12.66 nJ/bit is chosen to represent the whole energy spent on transmission. Therefore, $\gamma_{n}^{l}=12.66+\left\{2,3\right\}\times 2$ nJ/bit for n∈C (2 or 3 depending on the logical distance between *l* and the switch with the WAN connection).

### 5.2. Baseline/Suboptimal Solutions

To test the effectiveness of the proposed algorithm (*Full Optimization*, shortened on plots to *Full Optim*), we compare it with four simpler task allocation methods. A summary of these methods is shown in Table 5.

*Exhaustive Search*—all possible variations of allocations are verified. While this baseline approach finds the optimal solution, its running time scales exponentially with the number of requests. The optimal frequencies of CPU are calculated as in *Full Optimization*.

*Cloud Only*—all requests are transmitted to and processed in the cloud tier of the network. The optimal transmission allocation is obtained using a simplified version of the *Full Optimization*.

*No Migrate*—the nodes in the fog tier and cloud tier of the network cannot transmit tasks between themselves, i.e., the FN to which the request *r* is sent from the MD is the one that computes it ($a_{n}^{r}w_{l}^{r}=1\Leftrightarrow l=n$).

*Closest Wireless*—in this approach, requests are always transmitted wirelessly to the closest node (the one with the lowest path loss). Then, the rest of the optimization is performed as in *Full Optimization*. The difference lies mostly in the step described in Equation (33)—in Full Optimization the set of allocation variables w is found to minimize total transmission + computation costs, while in *Closest Wireless* each $w_{l}^{r}$ is found separately, minimizing “only” the wireless transmission costs.

Not all of these solutions are plotted on every graph for clarity in this section. The results of *Closest Wireless* in many configurations overlap with the results of *Full Optimization*. In other words, the results of *Closest Wireless* are indistinguishable (within 0.1%) from the optimal results of *Full Optimization* for the vast majority of tested parameter setups. Therefore, they are omitted from all plots except Figure 7, where the difference between these two is visible. Shaded areas around results for each solution show 95% confidence intervals.

### 5.3. Comparison with Exhaustive Search and All Possible Allocations

**Figure 3:**
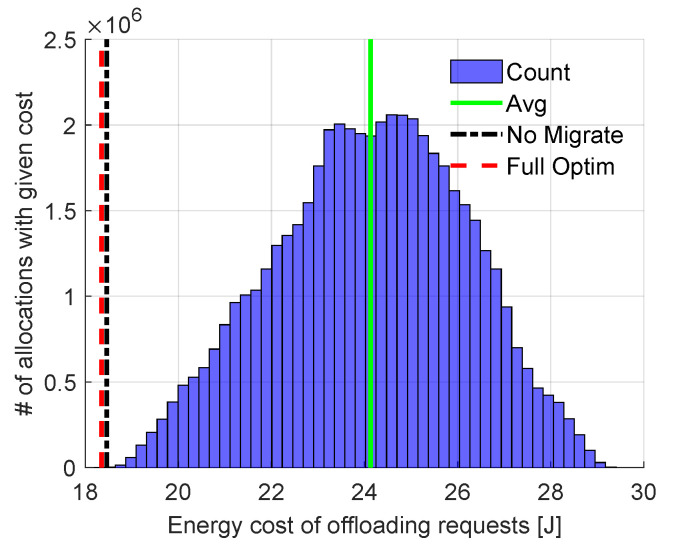
Comparison of our (Full Optim) solution with the No Migrate solution and all possible allocations from exhaustive search (blue bars; average value marked with solid green line).

First, let us compare results obtained from our *Full Optimization* with those resulting from *Exhaustive Search* to validate the ability of our algorithm to find the total minimum energy cost. A set of four computational requests is considered. The size of this set is limited due to the high computational complexity of *Exhaustive Search*. These requests have to be allocated among 10 FNs (allocation in the cloud is not considered in this example to highlight the importance of optimization within the fog tier). There are 50,400,000 possible allocations ($10^{4}$ for transmission, $\frac{10!}{(10-4)!}=5040$ for computation) in total with energy consumption varying from 18.3 J to more than 29.4 J, as presented in Figure 3. The results obtained by *Full Optimization* (red dashed line) and *No Migrate* (black dotted–dashed line) are also shown. *Full Optimization* does indeed find the same solution as *Exhaustive Search*. The solution found by *No Migrate* results in slightly higher energy cost. Still, both solutions provide energy cost significantly lower than the average cost of all possible allocations. It is clear that an algorithm which assigns requests to nodes randomly would not be efficient in terms of energy cost.

### 5.4. Impact of Network Parameters

**Figure 4:**
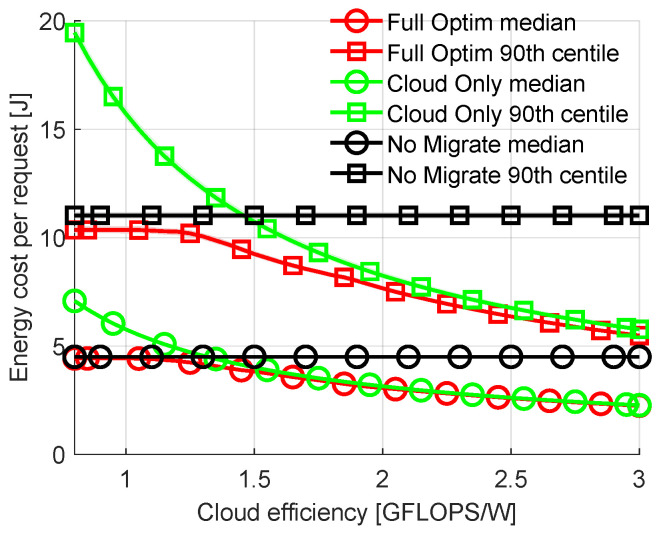
Comparison of energy cost per request with varied computational efficiency of cloud.

Now let us examine the impact of the computational efficiency of the cloud on energy costs and allocations in the full network. Let us sweep this efficiency from 0.8 to 3.0 GFLOPS/W (efficiency of the 500 most powerful commercially available computer clusters ranges from 0.19 GFLOPS/W to 39.4 GFLOPS/W with 4.04 GFLOPS/W as the median [35]). Figure 4 shows the median and the 90th percentile of the total energy costs spent on transmission and computation of offloaded requests. It can be seen that the energy costs of *Cloud Only* are significantly higher than those of *Full Optimization* for the lowest values of cloud efficiency, while differences between *No Migration* and *Full Optimization* are small. In all cases, our proposed solution requires a smaller amount of energy for a single request calculation than *No Migration*. As cloud efficiency increases, the cost of *Cloud Only* allocation decreases. In parallel, this allows *Full Optimization* to offload more tasks to the cloud, decreasing the energy consumption. The differences between the 90th percentiles are significantly higher than those between medians, showing the highest gains of *Full Optimization* for the most difficult requests. It is obvious that for the extremely high or low efficient cloud, the requests will be mostly calculated in the cloud or in the fog nodes, respectively. Therefore, for other results in this section, cloud efficiency is chosen to be 1.3 GFLOPS/W. This is a value of cloud efficiency that results in offloading decisions being not as straightforward as for values significantly higher or lower.

**Figure 5:**
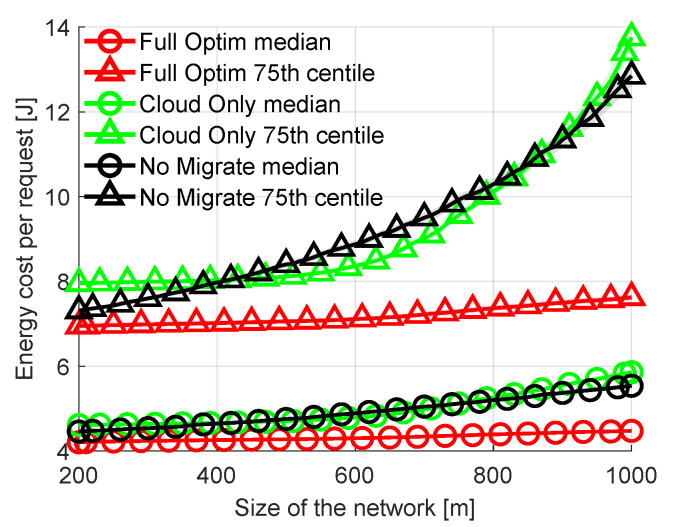
Comparison of energy consumption per request with varied size of area covered by the network.

Another network parameter that can impact the costs and offloading decisions is the physical size of the network. The network shown in Figure 2 (10 FN distributed over a 200m×50m hall) is used by default. Now let us vary the physical size of the network while maintaining the same number of FNs. This has an effect on the distance between MDs and FNs. The greater the distance, the higher the path loss and the energy-per-bit cost of wireless transmission. At the same time, the higher the path loss the lower the wireless transmission rate. In Figure 5 the length of the area covered by the network is swept up to 1000 m from the initial 200 m. With changing length (the longer of the two dimensions) the ratios of distances between all FNs and the area perimeter remain constant. The results in Figure 5 clearly show that the energy cost per request increases with the increasing size of the network. The increase is significant for *No Migrate* as MD is often “forced” to wirelessly send requests to more distant nodes if the close nodes are busy processing other requests or are not efficient enough. The rejection rates also increase from 3.3% at 200 m to 8.6% at 1000 m For *Full Optimization*, from 3.8% to 21.8% for *No migrate* and from 6.5% to 23.7% for *Cloud Only*. The difference in energy costs between *Full Optimization* and other methods becomes more apparent with increasing distances within the network.

### 5.5. Impact of Traffic Parameters

**Figure 6:**
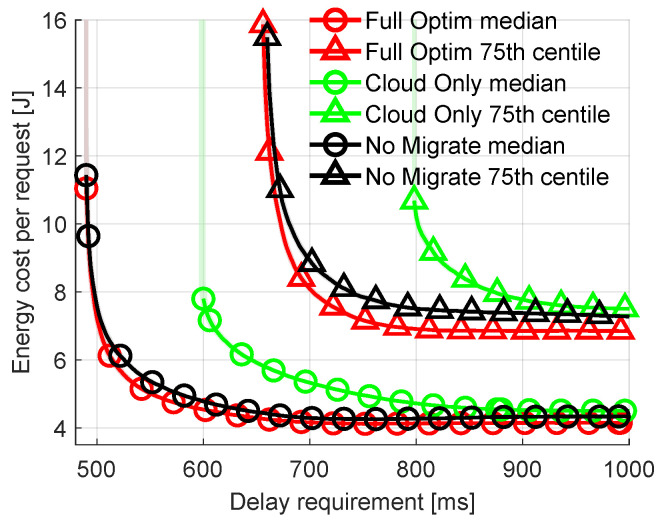
Comparison of energy consumption per request with varied delay requirement of requests.

Let us vary parameters characterizing the requests offloaded to the network. For previous results, the parameters characterizing offloaded requests are random, as shown in Table 4. First, let us look at the impact of the delay requirement. It is fixed for all the incoming requests. The other parameters (e.g., arrival rate, arithmetic intensity) are generated in the same way as described in Section 5.1. Figure 6 plots the median and the 75th percentile of energy costs spent on offloading requests as a function of the delay requirement (between 500 and 1000 ms) of these requests. There are a few key observations: (i) the percentage of rejected requests increases with stricter delay requirements, (ii) the energy cost increases with stricter delay requirements, (iii) *Cloud Only* is particularly poorly suited for delay-sensitive applications. Observation (i) is self-explanatory. The shorter the time-constraint, the harder it is to successfully offload the task, compute it and transmit the results back within this time. This can be seen on the plot where the respective lines terminate in the middle of a plot as a result of virtually infinite energy cost of a request that is unsuccessfully calculated. For example, the green line representing the 75th percentile of *Cloud Only* terminates at 800 ms. This means that for delay requirements lower than 800 ms more than 25% of requests are rejected. Observation (ii) is an effect of the higher CPU frequency required at the FN to fulfill stricter delay requirements. This results in decreased CPU efficiency and increased energy consumption. Observation (iii) stems from the additional transmission delay caused by sending requests to the distant cloud.

**Figure 7:**
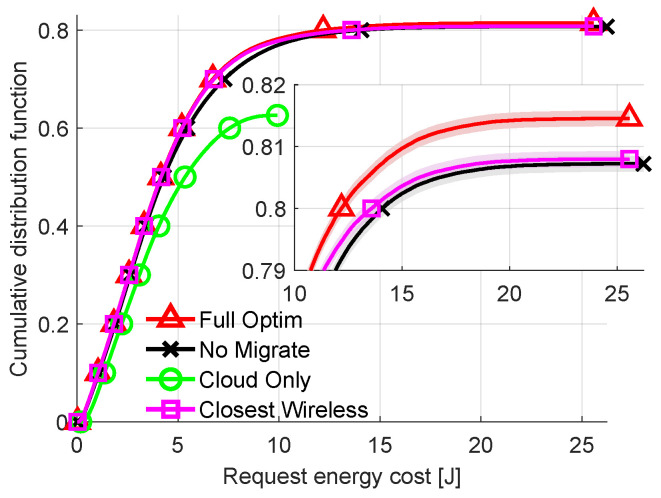
Comparison of energy consumption per request (CDF). Delay requirement: 700 ms.

To further analyze the difference between allocation strategies CDFs of energy costs are plotted in Figure 7 for fixed delay requirement of all requests equal to 700 ms. Unlike previous plots, Figure 7 includes results from the *Closest Wireless* algorithm. In all previous plots, the resulting energy costs of *Closest Wireless* are not shown, since they are either identical to those of *Full Optimization* or are within 0.1% of it. Lowering the delay requirement created a scenario where sending the request wirelessly to the nearest (cheapest) AP/FN and then finding the optimal node for computation may not result in the optimal solution. This shows that *Full Optimization* manages to successfully offload nearly 81% of all requests. This is the most out of all the compared methods, about 0.5 percentage point more than *Closest Wireless*. It is visible that all the methods are differentiated mostly for high percentiles of energy costs. The worst solution is *Cloud Only*, which rejects nearly 40% of all requests. While the difference between *Closest Wireless* and *Full Optimization* is relatively small, this can be treated as a promising suboptimal solution which decreases algorithm complexity while maintaining efficiency. This can change if the considered wireless technology, e.g., 5G NR, provides a higher data rate and higher energy efficiency. However, this requires energy consumption models of 5G terminals to be available.

**Figure 8:**
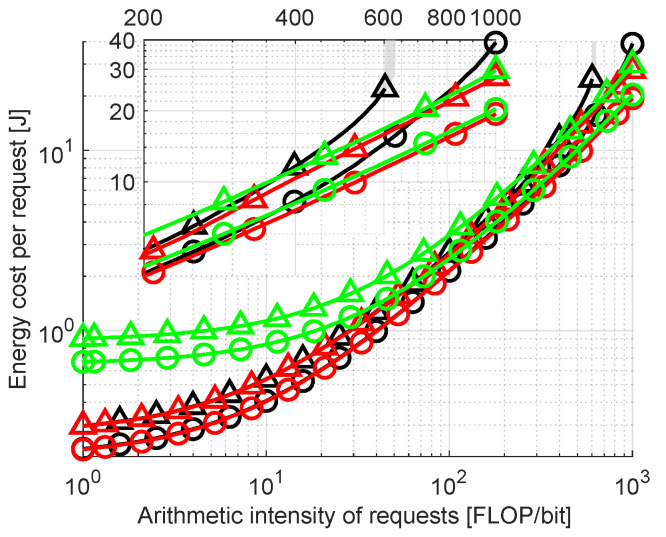
Comparison of energy consumption per request with varied arithmetic intensity. Same legend as in Figure 4 and Figure 5.

Finally, an impact of arithmetic intensity of offloaded requests is examined. This parameter determines how many computations are needed to process a given request relative to its size. The median and 75th percentile of energy costs for arithmetic intensity swept in range 〈1,1000〉 FLOP/bit are plotted in Figure 8. As expected, the energy cost increases with rising intensity. Higher values resulting from *Cloud Only* allocation at low intensity can be attributed to costs related to transmission (which do not directly depend on arithmetic intensity). Such requests can be more efficiently calculated in FNs, being commonly the result of the *Full Optimization* method. Energy costs (both median and 75th percentile) of *No Migrate* are within 10% of *Full Optimization* except for the values above 300 FLOP/bit where *No Migrate* steeply inclines. Rejection rates for *Full Optimization* are 1.1% for 1 FLOP/bit, 1.8% for 100 FLOP/bit and 11.7% for 1000 FLOP/bit. For *Cloud Only*, the values equal 2.9%, 4.4% and 20%, respectively. For *No Migrate*, they also start at 1.1% for 1 FLOP/bit and 1.8% for 100 FLOP/bit but reach 46.7% for 1000 FLOP/bit.

## 6. Discussion

We investigate the minimization of energy spent on offloading computational tasks in fog networks. Our model includes delay and energy costs resulting from computation as well as wireless and wired transmission. The proposed computational task allocation algorithm, *Full Optimization*, successfully minimizes energy consumption while satisfying delay constraints. All the considered degrees of freedom, i.e., AP selection, computing node selection and FN CPU frequency tuning increase system performance. However, precise gain characterization depends on a specific network configuration and specification of the computational requests. When compared with the *No Migrate* solution, the biggest performance improvements can be seen when offloaded tasks have high arithmetic intensity or when a large area covered by the network causes higher path loss (up to 50% lower energy consumption). Compared with performing all computations in the cloud, our solution is much better suited for requests with strict delay requirements and low arithmetic intensity. We also propose a heuristic approach that independently allocates wireless transmission called *Closest Wireless*. This simplified algorithm provides optimal solutions for almost all considered scenarios. Its performance is slightly worse for requests with strict delay requirements—it manages to satisfy delay constraints of 0.8% fewer requests compared to *Full Optimization* at 700 ms.

The limitations of this work include relying on energy consumption and delay models characterizing equipment in the network. Considering various devices available in the market, the models may not be accurate for all of them. Moreover, this work assumes some simplifications. Each request can only be computed at one node, while each FN can simultaneously process only one request. Future work includes extension of the setup with other wireless technologies, e.g., 5G NR. However, this requires reliable power consumption models for terminals of these technologies. Furthermore, metaheuristics targeting low execution times while finding close-to-optimal solutions may be an interesting research option. Another possible direction is adding a pricing mechanism to the network. This would incentivize FN and CN to prioritize processing certain requests and provide a price–delay trade-off.

## Figures and Tables

**Table 1:** Comparison with related works.

Energy (E) consumption and Delay (D)							
**Work**	**MD**	**MD-FN**	**FN**	**FN-FN**	**FN-CN**	**CN**	**Requests**
Dinh et al. [6]	Optim. incl. frequency	Optim.	Ign. E, Cons. D	N\A	Ign. E, Cons. D	Ign. E, Cons. D	Sets
You et al. [7]	Optim.	Optim.	Ign. E, Cons. D, One FN	N\A	Ign. E, Cons. D	Ign. E, Cons. D	Load
Liu et al. [8]	Optim.	Optim.	Ign. E, Cons. D	N\A	Ign. E, Cons. D	Ign. E, Cons. D	Load
Bai et al. [9]	Optim.	Optim.	Ign. E, Cons. D	N\A	Ign. E, Cons. D	Ign.	Sets
Vu et al. [10]	Optim.	Optim.	Ign. E, Cons. D	N\A	Ign. E, Cons. D	Ign. E, Cons. D	Sets
Deng et al. [11]	N\A	Ign.	Optim. incl. frequency	Ign.	Ign. E, Optim. D	Optim. incl. frequency	Load
Kopras et al. [12]	N\A	Cons.	Cons.	Ign.	Cons.	Cons.	Load
Vakilian et al. [13]	N\A	Ign. E, Cons. D	Optim.	Ign. E, Optim. D	Ign. E, Optim. D	Ign. E, Cons. D	Load
Khumalo et al. [14]	N\A	Optim.	Optim.	N\A	Ign. E, Cons. D	Ign. E, Cons. D	Load
Ghanavati et al. [15]	N\A	Optim.	Optim.	N\A	N\A	N\A	Sets
Sarkar et al. [16]	N\A	Cons.	Cons.	Ign.	Cons.	Cons.	Load
Kopras et al. [17]	N\A	Ign. E, Cons. D	Optim. incl. frequency	Optim.	Cons.	Cons.	Sets
This work	N\A	Optim.	Optim. incl. frequency	Optim.	Cons.	Cons.	Sets

**Table 4:** Simulation parameters.

Symbol	Value/Range	Symbol	Value/Range
**Requests**, $r\in R_{k}$			
$L^{r}$	[1, 5] MB	$\theta^{r}$	[1, 500] FLOP/bit
$o^{r}$	[0.01, 0.2]	$D_{\max}^{r}$	[500, 3000] ms
$\left|R_{k}\right|$	[5, 10]	$\bar{T_{k}-T_{k-1}}$	200 ms
**Computations in fog** [19,20,27], n∈F			
$p_{n,3}$, $p_{n,2}$	5.222, 34.256	$p_{n,1}$, $p_{n,0}$	88.594, −47.152
$f_{\min,n}$	1.6 GHz	$f_{\max,n}$	4.2 GHz
$s_{n}$	16 FLOP/cycle		
**Computations in cloud** [18,19], n∈C			
$f_{n}$	1.5 GHz	$s_{n}$	32 FLOP/cycle
**Wired Transmission** [23,24,28]			
$d^{n}$, n∈C	2000 km	χ	7500 ns/km
$b_{n}^{l},n\in C$	10 Gbps	$b_{n}^{l},n\in F$	1 Gbps
$\gamma_{n}^{l}$, n∈F	{2, 3} × 2 nJ/(bit)	$\gamma_{n}^{l}$, n∈C	12 nJ/bit
**Wireless Transmission** [29,30,31]			
$\gamma_{l}^{m^{r}}$, l∈F, $m^{r}\in M$	depends on rate and path loss	$b_{l}^{m^{r}}$, l∈F, $m^{r}\in M$	{0, 6.5, 13, 18.5, 26, 39, 52, 58.5, 65} Mbps

**Table 5:** Comparison of examined algorithms.

Name	Limitation	Optimization Variables
**Full Optimization**	None	Computing allocation—a, transmission allocation—w, computing frequency—f
**Exhaustive Search**	None	a, w, f
**Cloud Only**	$\sum_{n\in C}a_{n}^{r}=1,\;\forall r\in R_{k}$	w, a (if there are multiple Cloud Nodes)
**No Migrate**	$\sum_{l=n\in F}w_{l}^{r}a_{n}^{r}=1,\;\forall r\in R_{k}$	a interdependently on w, f
**Closest Wireless**	$w_{l}^{r}\;=\;\arg\min_{w_{l}^{r}}E_{\mathrm{wl}}^{r},\;\forall r\in R_{k}$	a, f

## Abbreviations

**AWS**	Amazon Web Services
**AP**	Access Point
**API**	Application Programming Interface
**BAN**	Body Area Network
**BS**	Base Station
**BSN**	Body Sensor Network
**CDN**	Content Delivery Network
**CDF**	Cumulative Distribution Function
**CPE**	Customer Premises Equipment
**CN**	Cloud Node
**CPU**	Central Processing Unit
**C-RAN**	Cloud Radio Access Network
**DC**	Data Center
**DVFS**	Dynamic Voltage and Frequency Scaling
**EA**	Energy-Aware
**ECG**	Electrocardiogram
**EEFFRA**	Energy-EFFicient Resource Allocation
**EH**	Energy Harvesting
**ETSI**	European Telecommunications Standards Institute
**EPON**	Ethernet Passive Optical Network
**FI**	Fog Instance
**FLOP**	Floating Point Operation
**FLOPS**	Floating Point Operations per Second
**FN**	Fog Node
**F-RAN**	Fog Radio Access Network
**GPS**	Global Positioning System
**GSM**	Global System for Mobile communications
**HD**	High Definition
**HT**	Higher Throughput
**IBStC**	If Busy Send to Cloud
**IBKiF**	If Busy Keep in FN
**IBStOF**	If Busy Send to Other FN
**IEEE**	Institute of Electrical and Electronics Engineers
**ICT**	Information and Communication Technology
**IoE**	Internet of Everything
**IoT**	Internet of Things
**IP**	Internet Protocol
**KKT**	Karush–Kuhn–Tucker
**LAN**	Local Area Network
**LC**	Low-Complexity
**LTE**	Long Term Evolution
**MCC**	Mobile Cloud Computing
**MINLP**	Mixed Integer Nonlinear Programming
**MD**	Mobile Device
**MEC**	Mobile/Multi-Access Edge Computing
**MIB**	Management Interface Base
**nDC**	nano Data Center
**NFV**	Network Function Virtualization
**NR**	New Radio
**OSI**	Open Systems Interconnection
**PA**	Power-Aware
**PC**	Personal Computer
**PGN**	Portable Game Notation
**QoE**	Quality of Experience
**QoS**	Quality of Service
**RAM**	Random Access Memory
**RAN**	Radio Access Network
**RFC**	Request for Comments
**RRH**	Remote Radio Head
**RTT**	Round-Trip Time
**SCA**	Successive Convex Approximation
**SCN**	Small Cell Network
**SDN**	Software Defined Network
**SDR**	SemiDefinite Relaxation
**SINR**	Signal to Interference-plus-Noise Ratio
**SM**	Sleep Mode
**SOA**	Service Oriented Architecture
**TDM**	Time Division Multiplexing
**TE**	Traffic Engineering
**TM**	Traffic Matrix
**TM**	Traffic Matrices
**V2V**	Vehicle-to-Vehicle
**V2X**	Vehicle-to-Anything
**VC**	Virtual Cluster
**VM**	Virtual Machine
**WAN**	Wide Area Network
**WDM**	Wavelength Division Multiplexing
**WE**	WeekEnd day
**WLAN**	Wireless Local Area Network
